# Optically Controlled Gain Modulation for Microwave Metasurface Antennas

**DOI:** 10.3390/s24061911

**Published:** 2024-03-16

**Authors:** Charlotte Tripon-Canseliet, Cristian Della Giovampaola, Nicolas Pavy, Jean Chazelas, Stefano Maci

**Affiliations:** 1 LPEM-CNRS, PSL, Sorbonne University, 75005 Paris, France; 2 ULTIMETAS, 75015 Paris, France; jean.chazelas@ultimetas.fr; 3 WAVE-UP, 53100 Siena, Italy; cdg@wave-up.it; 4 ESIEE, 93162 Noisy-Le-Grand, France; nicolas.pavy@esiee.fr; 5 Department of Information Engineering and Mathematics, UNISI, 53100 Siena, Italy; macis@dii.unisi.it

**Keywords:** metasurfaces, microwave antenna, light-matter interactions, photoconductivity, interferences

## Abstract

Over the past decade, metasurfaces (MTSs) have emerged as a highly promising platform for the development of next-generation, miniaturized, planar devices across a wide spectrum of microwave frequencies. Among their various applications, the concept of MTS-based antennas, particularly those that are based on surface wave excitation, represents a groundbreaking advancement with significant implications for communication technologies. However, existing literature primarily focuses on MTS configurations printed on traditional substrates, largely overlooking the potential benefits of employing photosensitive substrates. This paper endeavors to pioneer this novel path. We present a specialized design of a modulated MTS printed on a silicon substrate, which acts as a photosensitive Ka-band surface wave antenna. Remarkably, the gain of this antenna can be time-modulated, achieving a variance of up to 15 dB, under low-power (below 1 W/cm²) optical illumination at a wavelength of 971 nm. This innovative approach positions the antenna as a direct transducer, capable of converting an optically modulated signal into a microwave-modulated radiated signal, thus offering a new dimension in antenna technology and functionality.

## 1. Introduction

Metasurfaces (MTSs) are artificial, ultra-thin, two-dimensional structures with deep sub-wavelength thickness and electrically small unit cells; they can be engineered to control the interaction with electromagnetic fields. By carefully arranging these elements, metasurfaces can achieve remarkable control over electromagnetic (EM) waves, enabling functions such as anomalous reflection and refraction [1,2,3,4,5,6], polarization conversion [7,8], and beam-splitting [9,10]. Furthermore, MTS technology facilitates the conversion of surface waves (SWs) into space waves (SPWS) and vice versa [11,12,13,14,15], giving rise to a novel class of leaky-wave (LW) antennas characterized by high efficiency and customizable radiation patterns [16,17,18,19,20,21,22,23,24,25].

As for metamaterials (MTMs), the electromagnetic properties of MTSs can be effectively described in terms of homogenized parameters, thanks to the sub-wavelength size of the unit cell. However, unlike conventional metamaterials (MTMs), the thin layer thickness of metasurfaces dictates that their most suitable representation is not based on bulk constitutive parameters, but on boundary conditions (BCs) that interrelate the transverse components of the electromagnetic (EM) fields. In the microwave range, metasurfaces are commonly implemented using printed circuit board (PCB) technology, where electrically small metallic elements are printed over a dielectric slab [26]. In PCB technology, the electromagnetic properties of MTSs are controlled by patterning the metallic layer(s). These layers are represented using a penetrable equivalent impedance model. Reconfigurability of metasurface antennas represents an emerging field that involves modifying the equivalent impedance by electronic components, as well as reconfiguring the substrate with external electrical or optical parameters. This approach adjusts the complex permittivity of the substrate. However, existing literature primarily focuses on MTS configurations printed on traditional substrates, largely overlooking the potential benefits of employing photosensitive substrates. This paper endeavors to pioneer this method.

Printing MTS antennas on a silicon substrate allows for changing the complex permittivity, therefore obtaining a modification of antenna parameters, like the gain. Although the basic phenomenon of changing the complex permittivity in photosensitive material like silicon is well established, its implementation in MTS antennas represents a novel and promising path of exploration, opening up many application possibilities. This work demonstrates for the first time that the antenna’s gain can be significantly adjusted using a moderate amount of optical power. Such a technique enables the modulation of the radiated signal by directly injecting into the antenna’s substrate the optical modulated power. This direct modulation bypasses the need for electronic-to-optical or optical-to-electronic conversion systems, which typically introduce additional complexities, costs, and potential inefficiency. This ability suggests a high degree of dynamic reconfiguration compared to other Ka-band antenna technologies [27,28] (Table 1). This means that the antenna’s operational characteristics can be adjusted with extremely high speed, almost in real-time, to suit varying conditions or channel communication needs. This can translate into better communication quality and more reliable wireless connections. In this paper, we will not focus on optimizing the insertion of optical power into the antenna. Instead, our objective is to experimentally observe and physically interpret the underlying effectiveness of the basic physical phenomenon. To this end, the presentation of the paper is articulated as follows. Section 2 illustrates the photoconductive principle and the way to retrieve the complex permittivity. Section 3 focuses on the fabrication techniques of the MTS antenna printed on a silicon substrate.

## 2. Photoconductive Principle

The photoconductive effect is a physical phenomenon observed in semiconducting materials when they are exposed to optical illumination. In this condition, the material undergoes photogeneration of electron–hole pairs, which leads to a local change in conductivity. The light/matter interaction process controls complex material permittivity, and involves different parameters, such as optical power density, and wavelength, and charge carrier dynamics, such as their lifetime and mobility [34,35,36,37,38,39,40,41,42]. The general distribution of photoconductivity can be described as well from the superposition of the absorption parameter and the diffusion term, according to Platte and Sauerer [43]. The global approximation can be used to directly incorporate material absorption α, surface recombination velocity of photogenerated carriers $v_{s}$, their lifetime τ, and their ambipolar diffusion length L (1). The maximum photoconductivity $\Delta \sigma_{m}$ is given by (2) and the effective plasma depth $d_{e}$ by (3).

(1) $$L=\sqrt{\frac{2k_{B}.T\tau }{q}\frac{\mu_{n}.\mu_{p}}{\mu_{n}+\mu_{p}}}$$

(2) $$\Delta \sigma_{m}=\frac{\Delta \sigma_{0}}{1+\alpha L}{\left(\frac{1}{\alpha L}\frac{\alpha L^{2}+v_{s}\tau }{L+v_{s}\tau }\right)}^{\frac{-\alpha L}{1-\alpha L}}$$

and

(3) $$d_{e}=\frac{1}{\alpha }\left(\frac{L.(1+\alpha L+v_{s}\tau )}{L+v_{s}\tau }\right){\left(\frac{1}{\alpha L}\frac{\alpha L^{2}+v_{s}\tau }{L+v_{s}\tau }\right)}^{\frac{\alpha L}{1-\alpha L}}$$

Under optical illumination with energy superior to the band gap, Eg, free carriers, either electrons or holes, are photogenerated within the material in the illuminated area, changing its conductivity locally by a value of Δσ [35,44,45,46,47]. Extraction of the permittivity parameter for a reference high-resistivity silicon substrate can be performed by a Drude–Lorentz model, namely

(4) $$\varepsilon \left(\omega \right)=\varepsilon^{\prime }\left(\omega \right)+i\varepsilon^{\prime \prime }\left(\omega \right)$$

(5) $$\epsilon^{\prime }\left(\omega \right)=\epsilon_{s}+\frac{\sigma_{0}\tau }{\epsilon_{0}\left(1+\omega^{2}\tau^{2}\right)}$$

(6) $$\epsilon^{\prime \prime }\left(\omega \right)=-\frac{\sigma_{0}}{\omega \epsilon_{0}\left(1+\omega^{2}\tau^{2}\right)}$$

(7) $$\epsilon \left(\omega \right)=\epsilon_{s}-i\frac{\sigma (\omega )}{\omega \epsilon_{0}}$$

(8) $$\sigma \left(\omega \right)=\frac{\sigma_{0}}{1-i\omega \tau }$$

(9) $$\sigma_{0}=\tau .q^{2}\left(\frac{n}{m_{e}^{*}}+\frac{p}{m_{h}^{*}}\right)$$

where ω is the angular frequency, $\sigma_{0}$ represents the DC material conductivity, *n* denotes the concentration of conduction electrons, *q* denotes the electron charge, and $m_{e,h}^{*}$ denote the electron and hole effective masses, respectively. The following equations present the complex conductivity:

(10) $$\sigma \left(\omega \right)=\sigma^{\prime }\left(\omega \right)+i\sigma^{\prime \prime }\left(\omega \right)$$

(11) $$\epsilon \left(\omega \right)=\epsilon_{s}+\frac{\sigma^{\prime \prime }\left(\omega \right)}{\omega \epsilon_{0}}-i\frac{\sigma^{\prime }\left(\omega \right)}{\omega \epsilon_{0}}$$

with

(12) $$\sigma^{\prime }\left(\omega \right)=\frac{\sigma_{0}}{1+\omega^{2}\tau^{2}}$$

and

(13) $$\sigma^{\prime \prime }\left(\omega \right)=\frac{\omega \tau \sigma_{0}}{1+\omega^{2}\tau^{2}}$$

The photoinduced microwave complex permittivity of a highly resistive single-crystal silicon wafer can be extracted by the use of these formulas from a bistatic free-space characterization test bench operating in the Ka-band under CW optical illumination at wavelengths of 806 and 971 nm. The characterization test bench is illustrated in [48]

## 3. Ka-Band Metasurface Antenna on Silicon Substrate

Here, we outline the fabrication process of a silicon-based Ka-band MTS (metamaterial surface) antenna, which operates on the principle of surface wave excitation. The chosen material allows for the control of the microwave complex photoconductivity of the silicon substrate via laser beam illumination, leading to the consequent modulation of the antenna’s gain.

### 3.1. Fabrication of the Metasurface on Silicon

**Figure 1:**
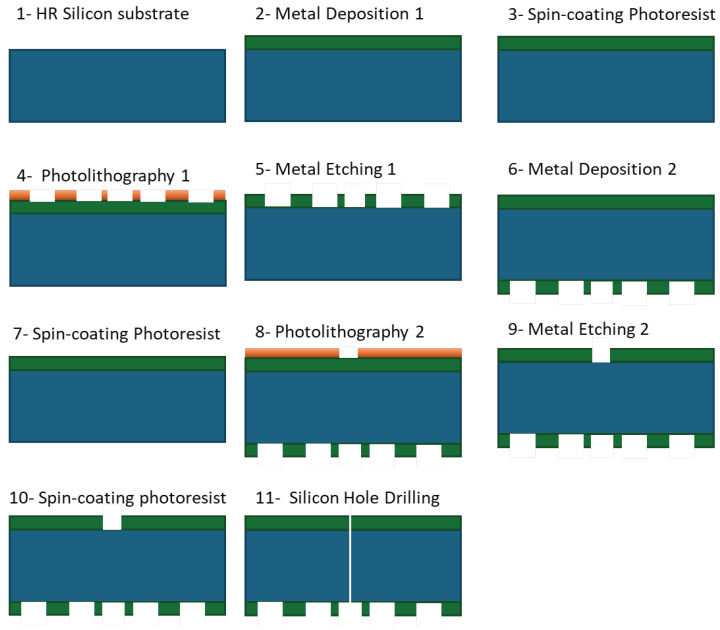
Step-by-step photolithography fabrication of the 4-inch metasurface antenna on the hi-res Si substrate.

The MTS antenna is fabricated on a 4-inch, 300 µm thick, high-resistivity silicon (hi-res Si) wafer. The substrate possesses a real part of the permittivity of 11.7. The fabrication process is delineated into nine distinct steps, as depicted in Figure 1. Initially, the first layer of metal is deposited on the Si substrate. Following this, a spin coating operation is performed to apply a thin, uniform layer of photoresist. Subsequently, a photolithography mask is applied, setting the stage for the etching of the metal. This step is succeeded by the deposition of a second metal layer at the antenna’s back surface. Another photolithography mask is then applied, preceding the etching of the ground-plane metal layer. The process culminates with the etching of silicon to create the central hole, completing the fabrication sequence. This hole facilitates the passage of the internal conductor of a coaxial probe, ultimately becoming a monopole antenna responsible for exciting the surface wave on the metasurface.

### 3.2. Antenna Layout

**Figure 2:**
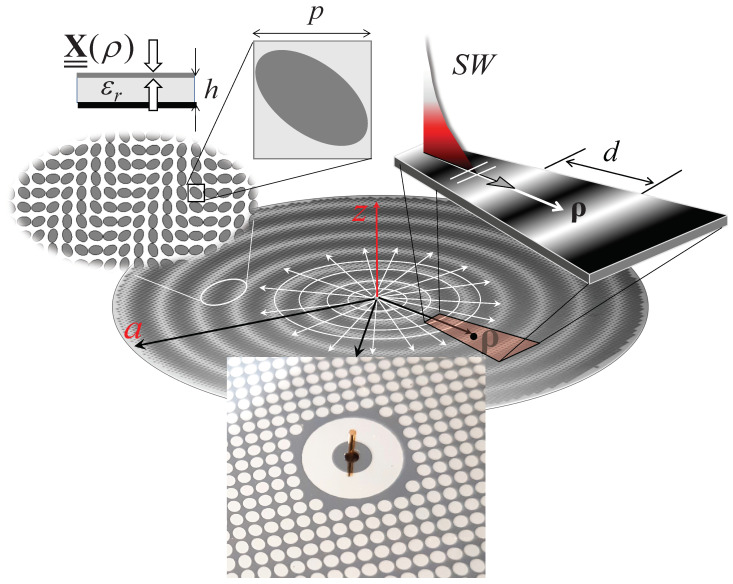
Physical mechanism for the MTS antenna based on surface wave excitation. Top inset: elliptical printed patch layout and basic periodic cell. Bottom inset: an image of the central part of the realized antenna with the monopole feed.

The MTS antennas are designed for operation at 30 GHz. The printed area of the pattern has a circular area with a 10 cm diameter, occupying almost the entire 4-inch silicon wafer. The printed texture is composed of sub-wavelength elliptical metal patches, accommodated in a regular lattice with a period of p=1.5 mm = λ/6.6. The layout is depicted in Figure 2. The lower inset of this figure shows an image of a part of the realized antenna, which includes the monopole feed, placed at the center of the aperture. This feed is comprised of a circular ring featuring a central hole that tunnels through the substrate. A coaxial V-type connector is used for feeding; the internal conductor of the coaxial is utilized to form a monopole above the substrate, threading through the hole. This arrangement is used to excite a cylindrical surface wave on the substrate. This surface wave, interacting with the modulated MTS texture, generates a leaky wave associated with a circularly polarized broadside beam. For this purpose, the design of the equivalent anisotropic reactance follows the synthesis method proposed in [18]. Specifically, the dimensions and orientation of the elliptical patch elements are configured to achieve an anisotropic reactance with periodic modulation *d* in the radial direction (Figure 2). This period matches the wavelength of the surface wave supported by the average value reactance. The combination of this modulation with a linear azimuth phase and a particular relationship with the entries of the anisotropic tensor results in the generation of a circularly polarized beam at the broadside.

## 4. Numerical Model

**Figure 3:**
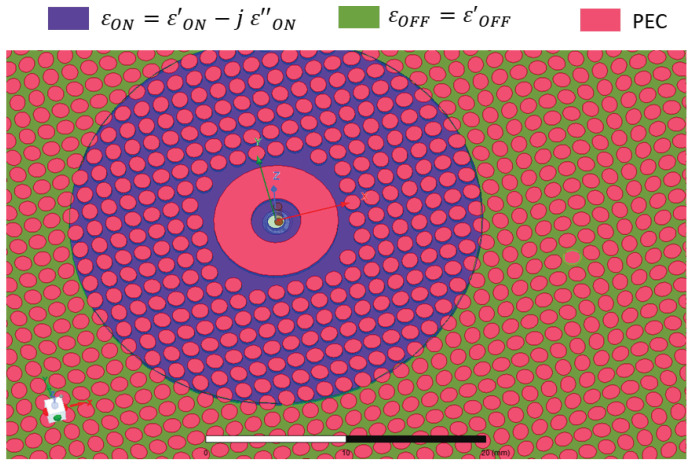
Top view of the numerical model. The metallic area is shown in orange; the background shows two different colors. Green denotes the area with a relative permittivity of 11.51, not illuminated by the optical beam. Purple denotes the area with complex permittivity, retrieved by measurements with the procedure outlined in Section 2. This complex permittivity models the illuminated area.

A full-wave analysis was carried out by ANSYS HFSS Software. The numerical layout is shown in Figure 3.

**Figure 4:**
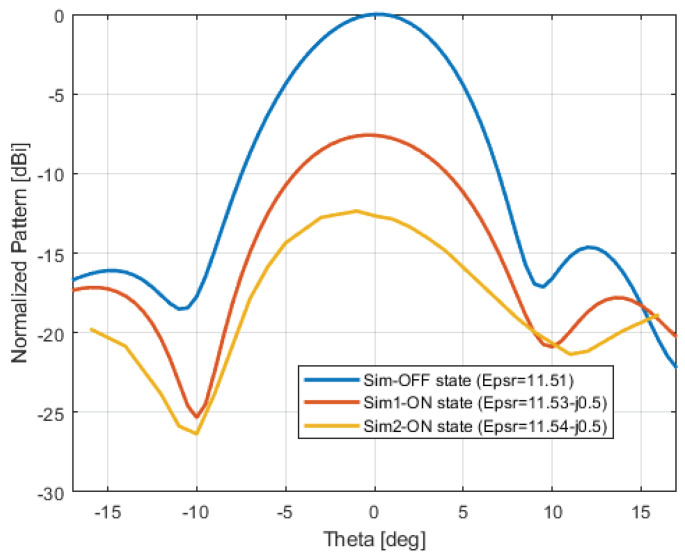
Full-wave simulation relevant to the normalized radiation pattern in the OFF state and with two different values of the complex permittivity retrieved by the measurements.

A simulation in the OFF state (namely, with no illumination) was carried out by assuming a silicon dielectric constant of 11.51 without an imaginary part. The ON state was simulated by assuming a complex dielectric constant in a circular area that was illuminated by the laser beam (purple background in Figure 3). The numerical results of normalized radiation patterns are shown in Figure 4. Complex dielectric constants, shown in this figure, are retrieved by the formulas described in Section 2 and the measurements presented in [48], based on the optical power density (OPD) and optical wavelength specified in the next section.

## 5. Optical Control and Antenna Gain Modulation

### 5.1. Experimental Setup and Measurements

**Figure 5:**
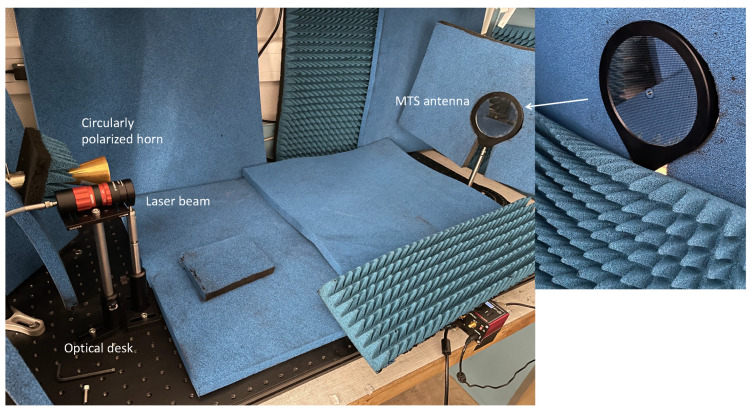
Experimental setup.

The antenna radiation pattern was characterized in an anechoic environment using a Rohde & Schwarz vector network analyzer operating in the 26.5–40 GHz frequency range. The setup is shown in Figure 5. A calibrated circularly polarized horn antenna was used. Azimuth radiation patterns were achieved at this frequency by placing the device on a high-precision rotating stage, under both dark and laser-based optical illumination conditions.

The lighting conditions were achieved using a high-power, fiber-coupled laser source equipped with a collimation system. This setup enables targeted illumination on the front side of the antenna, allowing the adjustment of the OPD and the optical wavelength. Specifically, at a wavelength of 971 nm, the laser’s optical power was arranged to reach a peak OPD of 0.8 W/cm², concentrated within an optical beam waist diameter of 4 cm.

**Figure 6:**
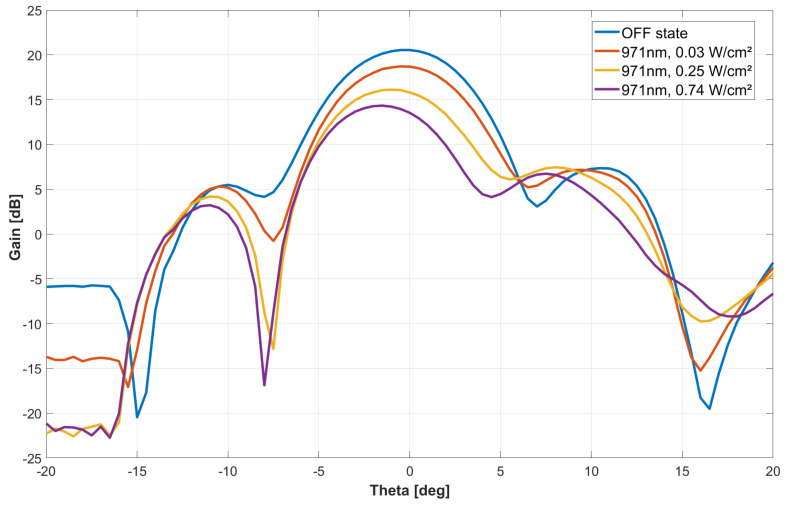
Measured azimuthal RHCP Si-based MTS antenna radiation pattern at 29.5 GHz under 971 nm of wavelength optical control at various power densities.

**Figure 7:**
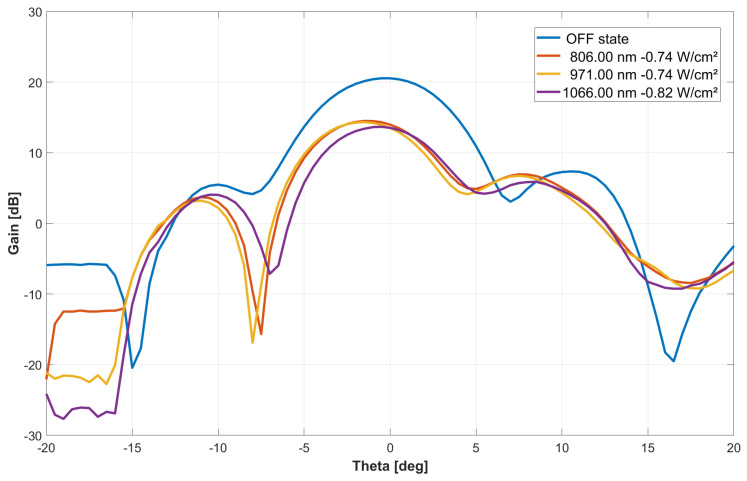
Azimuthal RHCP Si-based MTS antenna radiation pattern at 29.5 GHz of microwave frequency under different optical wavelengths with a power density of 0.74–0.82 W/cm².

Under these conditions, at an optical wavelength of 971 nm, the MTS antenna undergoes a variable light-dependent gain performance. A broadside antenna gain reduction was achieved as a function of the optical power density in a range of 10 dB (Figure 6). Specific data are summarized in Table 2. At the same time, an angle deviation of a few degrees is observed for the main lobe’s maximum radiation, which can be interpreted from substrate material complex permittivity changes during illumination. The same characterization procedure was executed at different optical wavelengths in order to check the antenna’s performance sensitivity to this parameter (Figure 7 and Table 3). This phenomenon is also attributed to variations in the penetration depth of the HR silicon material with optical wavelength, which, in turn, influences the imaginary part of its precipitation.

### 5.2. Interpretation in Terms of Antenna Efficiency Contributions

When the illumination is applied on the topside of the metasurface antenna, the light penetrates inside the opened silicon area, modifying its local complex permittivity.

Both the real and the imaginary parts of the permittivity affect the antenna efficiency and, therefore, its gain. The real part of the permittivity of the substrate material affects the propagation constant of the SW launched by the feed, inducing a mismatch between the SW wavelength and the period of the impedance modulation; this changes the mechanism of energy transfer between the surface wave and leaky wave, creating feed mismatching and radiation pattern alterations. The imaginary part of the permittivity induced by light produces an increase in Ohmic losses. Referring to the description and terminology in [49], the feed efficiency, $\epsilon_{feed}$, is given in (14), where $P_{feed}$ is the space wave power radiation by the feed, which decreases due to light absorption. At the same time, the constant propagation, $\beta_{sw}$, of the surface wave launched from the feed is modified by the light intensity and mismatched to the metasurface modulated impedance, leading to a change in the conversion efficiency, $\epsilon_{conv}$, given in (15). The latter efficiency accounts for the power transformation from the SW to the leaky wave (LW), and it is defined as the power $P_{lw}$ radiated by the surface leakage in the absence of losses and the surface wave power, $P_{sw}$, launched by the feed. We also note that part of the SW power is dissipated in ohmic losses, $P_{\Omega }$, and, therefore, is not radiated by the LW mechanism; this can be accounted for by further multiplicative loss efficiency, $\epsilon_{\Omega }$ in (16).

(14) $$\epsilon_{feed}=1-\frac{P_{feed}}{P_{in}}$$

(15) $$\epsilon_{conv}=\frac{P_{lw}{|}_{P_{\Omega }=0}}{P_{sw}}$$

(16) $$\epsilon_{\Omega }=\frac{P_{lw}}{P_{lw}{|}_{P_{\Omega }=0}}$$

(17) $$\epsilon_{tap}=\frac{\lambda^{2}}{4\pi }.\frac{1}{A}.S_{max}.\frac{4\pi .r^{2}}{P_{lw}}$$

A further tapering efficiency as in (17) is defined to relate the effective area to the physical area due to the tapering illumination. In (17), *A* and *r* are the antenna aperture and radius, and $S_{max}$ is the maximum amplitude of the Poynting vector at a distance, *r*, in the far zone of the antenna. The overall antenna gain results in $G={\left(ka\right)}^{2}$$\epsilon_{feed}\epsilon_{conv}\epsilon_{tap}\epsilon_{\Omega }$. The magnitude of the reflection coefficient at the input of the antenna remains at a value of −10 dB in the frequency band, indicating that the absorption of light inside the substrate does not significantly affect the antenna’s input matching and accepted gain.

## 6. Antenna Input Impedance

**Figure 8:**
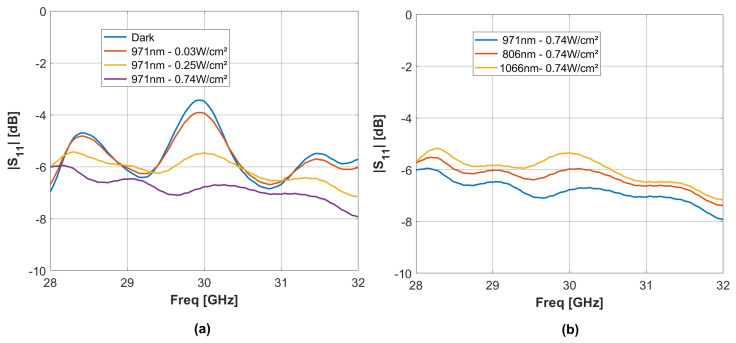
The measured $S_{11}$ reflection parameters of the Si-based MTS antenna between 28 and 32 GHz: (**a**) under laser-based illuminations at different OPDs and constant wavelengths—(**b**) under laser-based illuminations at different wavelengths.

Complex input reflection coefficient $\left(S_{11}\right)$ measurements at the antenna port were also performed in order to validate the impedance matching at 29.5 GHz as a function of the optical power. The initial set of measurements focused on comparing the magnitude of the $S_{11}$ antenna reflection coefficient under two conditions: in a dark environment and under laser-based illumination at 971 nm with different OPD (see Figure 8). These results reveal two primary physical effects. The first pertains to the central hole diameter, which exceeds that of the connector pin (1 mm vs. 600 µm), causing a capacitive effect that slightly influences the matching frequency bandwidth at a level of −10 dB. Indeed, at 29.85 GHz, the extracted complex antenna input impedance $Z_{in}=Z_{0}\left(1+S_{11}\right)/\left(1-S_{11}\right)$ is equal to 25 Ω–j40 Ω, which remains quasi-constant during illumination. This effect is mainly constrained by the technological process of the hole. The second one is related to the substrate permittivity change under illumination, which only affects antenna radiation by gain reduction while improving input antenna matching to 50 Ω during optical illumination.

The second set of measurements was devoted to comparing the antenna reflection coefficient under different laser-based illumination wavelengths and the same OPD (Figure 8), namely 806 nm, 971 nm, and 1066 nm. These variations are due to the change in permittivity caused by the different light penetration depths at different optical wavelengths. This was also found in [48].

## 7. Conclusions and Discussion

A metasurface-based antenna was developed and constructed on a highly resistive silicon substrate, designed specifically for using the photoconductive property. Characterization of the antenna in the Ka-band microwave range was conducted both in a dark environment and under near-infrared (NIR) laser illumination. The numerical results and experiments demonstrate that the optical control exerted on the metasurface antenna influences its overall efficiencies, reducing the antenna’s gain as a result of changes in the substrate’s permittivity, both in its real and imaginary components. Furthermore, the experiments confirm that illumination leads to a partial adjustment in the antenna’s surface impedance mismatch because of the permittivity alterations in the substrate, affecting the antenna’s radiation pattern while ensuring the maintenance of overall input antenna impedance matching. These outcomes provide a unique advantage for secure communications, as optical switching between ON and OFF states offers a means to transmit additional information by modulating the Ka-band signal directly with light.

## Figures and Tables

**Table 1:** Comparison of Ka-band flat antenna technologies.

Ref	Frequency (GHz)	Bandwidth (%)	Gain (dB)	Technology
[29]	28.6	-	24.8	PSWA
[30]	29.1–32.5	11.3	9	OERWA
[31]	34	35.5	17.4	SIWG
[32]	26.5–40	40	12.5	CSIW
[33]	28	13.92	14.4	PSP
this work	30	20	20	MTS

Planar slotted waveguide array (PSWA), open-ended rectangular waveguide antenna (OERWA), substrate integrated gap waveguide (SIWG), corrugated substrate integrated waveguide (CSIW), planar segmented patch (PSP), metasurface antenna (MTS).

**Table 2:** Main lobe gain reduction vs. optical power density @971 nm illumination.

Power Density (W/cm²)	Main Lobe Gain Reduction (dB)
0.03	2
0.25	4.6
0.74	6.8

**Table 3:** Main lobe gain reduction vs. the optical wavelengths with a power density of 0.74–0.82 W/cm².

Wavelength (nm)	Main Lobe Gain Reduction (dB)
808	6.7
971	6.8
1066	6.9

## Data Availability

The data presented in this study are available on request from the corresponding author due to associated private company policies.
